# Cyclic strength of imperfectly saturated sands and analysis of liquefaction

**Published:** 2004-09-01

**Authors:** Kenji Ishihara, Yoshimichi Tsukamoto

**Affiliations:** *) Professor of Civil Engineering, Chuo University, 1-13-27, Kasuga, Bunkyo-ku, Tokyo 112-8551, Japan; ***) Associate Professor of Civil Engineering, Tokyo University of Science, 2641, Yamazaki, Noda, Chiba 278-8510, Japan

**Keywords:** Liquefaction, earthquake, P-wave velocity, unsaturated sands

## Abstract

The simplified method of analysis to assess liquefaction potential of a given sand deposit is briefly introduced in the first part of this paper. Then, recent advances in the laboratory testing for evaluating liquefaction resistance are described with a particular emphasis on the sand partly saturated with water. As a means to identify the degree of saturation which is applicable for both field deposits and laboratory samples, the use of the longitudinal wave (P-wave) is proposed based on a suite of data obtained from the triaxial tests in the laboratory. It is recommended that the non-destructive P-wave measurements be carried out first to determine the degree of saturation, and then the cyclic triaxial tests involving large destructive strains should be performed to determine the cyclic strength on the same sample of the sand. In order to demonstrate usefulness of the proposed approach, two sets of undisturbed samples were secured from two sites; one is located in Sakai-minato city which has suffered severe liquefaction at the time of the 2000 Tottoriken-Seibu earthquake and the other site is located in Koshigaya city, Saitama, where likelihood of liquefaction to occur in a future earthquake is of major concern. Penetration tests and in-situ velocity logging were also conducted at these two sites. By adjusting the P-wave velocity of the undisturbed samples in the laboratory so as to have the same velocity in the field, the in-situ state of saturation was reproduced in the laboratory samples. Then, the cyclic loading tests were conducted to determine the cyclic strength of intact samples. The results of the laboratory tests as above were incorporated into the simple method of liquefaction analysis described in the first section of this paper. The analysis seems to yield results which are in reasonably good agreement with what was observed at the time of the earthquake.

## Introduction

In the design practice of foundations of structures, cohesionless soils such as gravel or sand have long been deemed as materials providing sound and stable bases. This fact proves valid for the environments in which loads are applied monotonically in a static manner. On the contrary, cohesive soils such as clay or silt have been the source of concern in a static load environment, because of their high compressibility and low resistance to shear stress application in relation to settlements or failure of the ground.

In the environments of dynamic loading in which loads are applied rapidly and repetitively, the circumstances as above are reversed with the cohesionless soils posing real problems, whereas cohesive soils behave rather favorably. The liquefaction of saturated loose sand undergoing the cyclic load during earthquakes is a typical example of poor performance of cohesionless soils. Since the subject of earthquake-induced liquefaction has been the main issue of concern among engineers in the seismic region, the present state-of-the-art on the simple method of liquefaction analysis will be briefly introduced first in this paper. Then, some facets of new development will be given regarding evaluation of cyclic resistance focusing on partly saturated sand and effects of saturation.

## Mechanism and definition of liquefaction

**Fig. 1:**
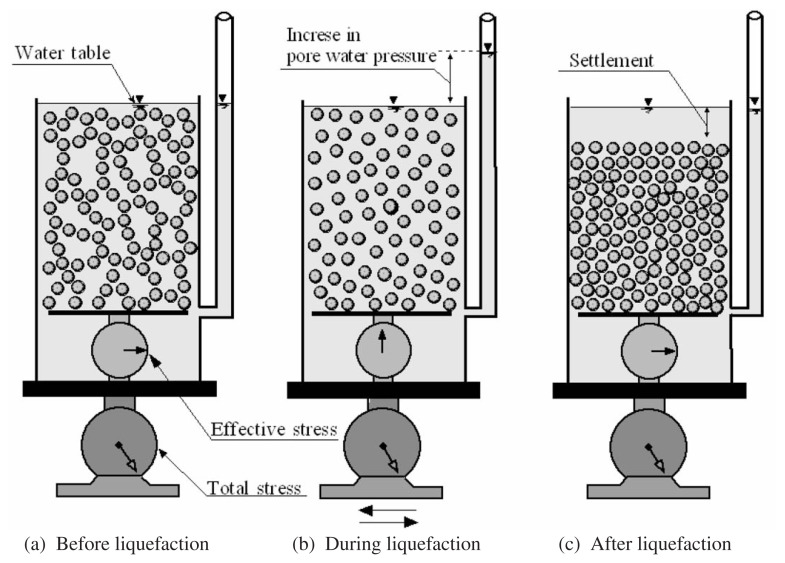
Transfer of state of deposition via liquefaction.

It is widely recognized that the basic mechanism of liquefaction in a deposit of loose saturated sand during earthquakes is the progressive build-up of excess pore water pressure due to the application of cyclic shear stresses induced by the upward propagation of shear waves from the underlying rock formation. Under ordinary conditions prior to an earthquake, a soil element in level ground is subjected to a confining stress due to the weight of the overlying soils, as schematically illustrated in Fig. 1(a). When a series of cyclic stress is applied during an earthquake, the element of the loose sand tends to reduce its volume. However, since the duration of the cyclic stress application is so short as compared to the time required for drainage of water towards the surface from deposits of several metres depth, the volume contraction cannot occur immediately. In order to keep the potentially contracting loose sand at a constant volume, some change in the existing stress system must take place. This stress change is achieved in the form of a reduction in the existing confining stress due to the overlying soil, accompanied by concurrent increase of equal magnitude of pore water pressure. Therefore, the degree of pore water pressure increase depends, on one hand, upon the looseness or denseness of sand deposits indicative of potential of the volume decrease tendency and, on the other hand, upon how largely the sand is sheared to extract the inherent volume decrease characteristics. When the state of sand packing is loose enough and the magnitude of cyclic shear stress is great enough, the pore water pressure will build up to a full extent in which it becomes equal to the initially existing confining stress. At this state, no effective stress or inter-granular stress is acting on the sand and individual particles released from any confinement exist as if they were floating in water, as schematically illustrated in Fig. 1 (b). Such a state is called liquefaction. Upon occurrence of liquefaction, individual particles of the sand start to sediment in water, thereby expelling pore water towards the surface of the deposit and when the sedimentation has taken place throughout the depth, the sand is now deposited in a somewhat denser state, as illustrated in Fig. 1(c). The transfer of the state of sand from the initial deposition to the final dense state via the process of liquefaction is illustrated schematically in Fig. 1, in which the scale inside the box is assumed to indicate the effective stress and the outside scale supporting the sand and water-filled box indicates the total stress.

**Fig. 2:**
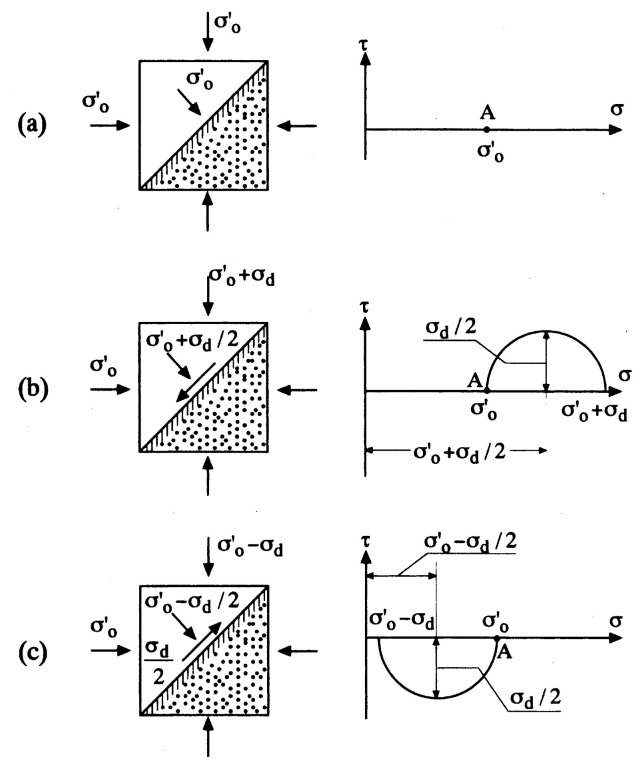
State of stress in the triaxial sample.

The above considerations are concerned with overall interpretation on the mechanism of liquefaction. A more in-depth understanding of the liquefaction phenomenon can be gained from observation of behaviour of a sand sample undergoing cyclic stress application which can be reproduced in the laboratory triaxial test apparatus. The sample of saturated sand is consolidated under a confining pressure and subjected to a sequence of constant-amplitude cyclic axial stress under undrained conditions, until it deforms to a certain amount of peak-to-peak axial strain. In the triaxial test conditions, the maximum shear stress is known to act on the plane inclined by 45˚ from the horizontal, as illustrated in Fig. 2(a). Therefore, the initial state of isotropic consolidation with a confining stress, ***σ***_o_’, is represented by point A in the Mohr stress circle. When the axial stress is increased by an amount, ***σ***_d_, the shear stress induced on the 45˚-plane is ***σ***_d_/2 and directed downwards to the left, as illustrated in Fig. 2(b). When the axial stress is reduced by an amount, ***σ***_d_, the resulting shear stress on the 45˚-plane is also ***σ***_d_/2 but its direction is reversed as shown in Fig. 2(c). From the above considerations, it is recognized that the amplitude of shear stress in the cyclic triaxial loading condition is represented by ***σ***_d_/2, and by normalizing this amplitude to the initial confining stress, the cyclic stress ratio can be defined as being ***σ***_d_’/(2***σ***_o_’). The cyclic stress ratio thus defined has been used to represent the relative magnitude of the external shear stress being applied to the soil samples in the triaxial test equipment.

**Fig. 3:**
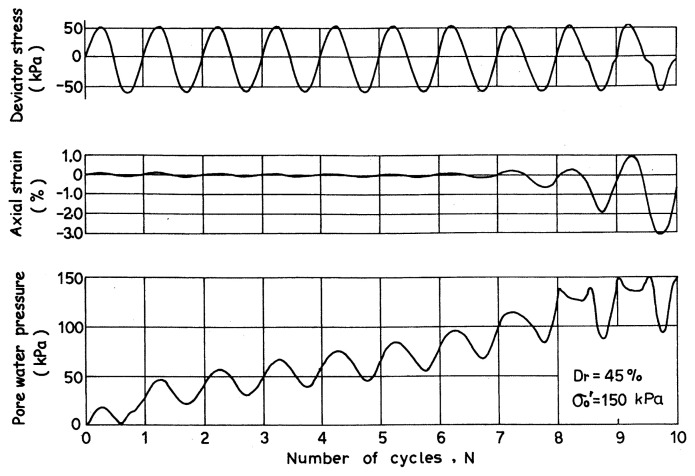
Records of cyclic triaxial test.

Typical results of cyclic triaxial test on a loose sample of Niigata sand are demonstrated in Fig. 3, where it may be seen that the pore water pressure builds up steadily as the cyclic stress is applied, and eventually approaches a value equal to the initially applied confining pressure, thereby producing an axial strain of about 5% in double amplitude. Such a state has been referred to as “initial liquefaction”. For loose sand, the initial liquefaction may be taken as a state of failure, because indefinitely large deformation is produced during a few cycles of load application following the initial liquefaction. For dense sand, about 5% double-amplitude axial strain is produced when the initial liquefaction occurs. However, the deformation after the initial liquefaction does not grow indefinitely large and complete failure does not take place in the sample even after the onset of initial liquefaction. Nonetheless, some degree of softening takes place in the dense sample accompanied by a sizable amount of cyclic strain and therefore it has been customary to consider the onset of initial liquefaction or the development of 5% double-amplitude axial strain as a measure to identify a state of cyclic instability for all densities of sand on an equal basis. In what follows, the 5% double-amplitude axial strain in the cyclic triaxial test will thus be taken up as a criterion to consistently define the state of initial liquefaction of any density of sand from loose to dense state.

**Fig. 4:**
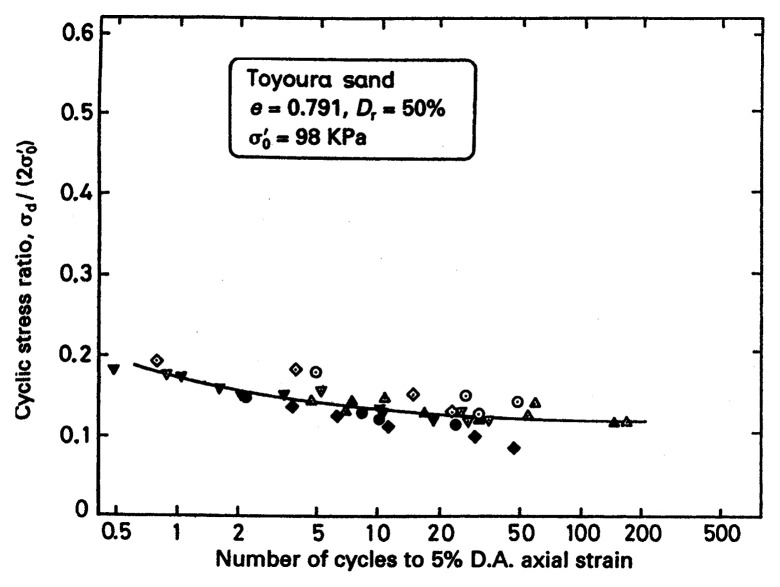
Results of the co-operative tests in Japan on the cyclic strength of sand (Toki *et al*. 1986).

In order to define the conditions of initial liquefaction or development of 5% double-amplitude axial strain, it is further necessary to specify the number of cycles in the constant-amplitude cyclic loading test. In principle, the number of cycles may be set as arbitrary, but is has been customary to consider 20 cycles to define the state of initial liquefaction. Thus, the condition of initial liquefaction can be specified in terms of the magnitude of cyclic stress ratio under 20 cycles of uniform load application. The cyclic stress ratio defined in this way is sometimes referred to simply as cyclic strength. Typical results of the cyclic triaxial tests on Toyoura sand (Japanese Standard sand) are shown in Fig. 4, (Toki *et al*. 1986),1) where the cyclic stress ratio causing 5% double amplitude of axial strain is plotted versus the number of cycles of load application. The data in Fig. 4 are the collection of results of tests from various laboratories, which were conducted under unified test procedures stipulated by the Japanese Society of Geotechnical Engineering. In this diagram, an average value of the cyclic strength for 20 cycles is read off as R = (***σ***_d_/2***σ***_o_’)_20_ = 0.15.

## Cyclic shear stress induced by motions of earthquakes

The shear stresses induced at any point in the level ground during an earthquake are due mainly to the upward propagation of shear waves in the deposit from an underlying rock formation. The shear stresses in the soil deposit at shallow depths where liquefaction is most liable to occur can be assessed by means of a simple procedure proposed by Seed and Idriss (1971).2)

**Fig. 5:**
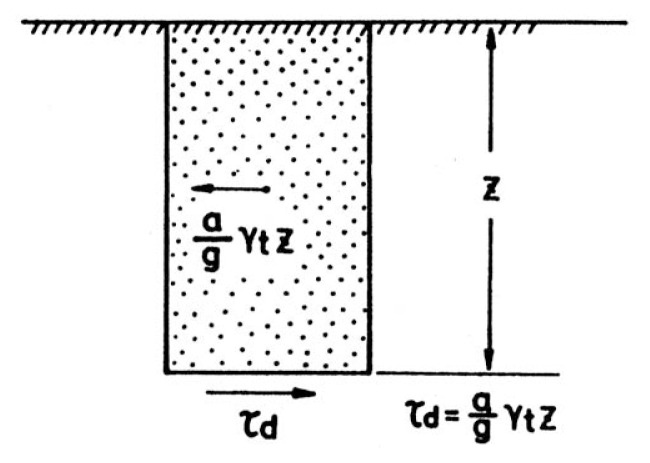
Relationship between the ground acceleration and the induced shear stress.

Consider a soil column to a depth, z, as shown in Fig. 5. If the soil column to a depth, z, is assumed to move horizontally as a rigid body and if the maximum horizontal acceleration on the ground surface is, a_max_, the maximum shear stress, ***τ***_max_, acting at the bottom of the soil column would be,

[1] $$\tau_{\text{max}}=\frac{{\text{a}}_{\text{max}}}{\text{g}}\cdot \gamma_{\text{t}}\cdot \text{z},$$

where ***γ***_t_ is the unit weight of the soil and g is the gravity acceleration. Since the soil column moves as a deformable body, the actual shear stress will be less than that given by Eq. [1] and might be expressed by,

[2] $$\tau_{\text{max}}=\frac{{\text{a}}_{\text{max}}}{\text{g}}\cdot {\text{r}}_{\text{d}}\cdot \gamma_{\text{t}}\cdot \text{z},$$

where r_d_ is a stress reduction coefficient which takes a value less than unity. Seed and Idriss (1971)2) expressed values of r_d_ in a graphical form but Iwasaki *et al*. (1978)3) subsequently recommended the use of the empirical formula,

[3] $${\text{r}}_{\text{d}}=1-0.015\text{z},$$

where z is in metre. By dividing both sides of Eq. [2] by the effective overburden stress, ***σ***_v_’, Eq. [2] is modified to read,

[4] $$\frac{\tau_{\text{max}}}{{\sigma_{\text{v}}}^{\prime }}=\frac{{\text{a}}_{\text{max}}}{\text{g}}\cdot {\text{r}}_{\text{d}}\cdot \frac{\sigma_{\text{v}}}{{\sigma_{\text{v}}}^{\prime }},$$

where ***σ***_v_ and ***σ***_v_’ denote the total and effective overburden stress, respectively. The above equation has been used widely to assess the magnitude of shear stress induced in a soil element during an earthquake. One of the advantages for using Eq. [4] is that the vast amount of information on the accelerations ever recorded on the ground surface can be used directly to assess the shear stresses in the ground.

It is apparent that the type of relation expressed by Eq. [4] can hold valid at any instant of time throughout the time duration of earthquake motions. This implies the fact that any time change in the shear stress in the soil deposit at shallow depths takes place in unison with time variation of the acceleration on the ground surface, the difference being only in the relative magnitude. Therefore, a time history of shear stress in the soil has the same general shape as the time history of acceleration at the ground surface.

## Cyclic resistance of sand in irregular seismic loading

Time histories in shear stress application due to upward propagation of shear waves through the level ground are essentially irregular and multi-directional when viewed on the horizontal plane. In order to quantitatively represent the liquefaction characteristics of sand in such a complicated loading environment, it has been a common practice to introduce some coefficients correcting for the cyclic strength under uniform cyclic loading to allow for the effects of multidirectional irregular nature of shear stress application during earthquakes. For this purpose, multiple series of laboratory tests have been carried out using a simple shear test equipment in which loads with various irregular time histories were applied to the specimens in two mutually perpendicular direction, (Ishihara and Nagase, 1985).4) By feeding two components of acceleration time histories recorded during actual earthquakes to the specimens prepared in this test device, the magnitude of irregular shear stress needed to produce a state of initial liquefaction in the simple shear specimens was determined. On the other hand, tests with uniform loading in one-direction only were conducted using the same simple shear test apparatus and the cyclic stress ratio causing 5% double-amplitude (DA) shear strain under 20 cycles of constant-amplitude cyclic stress application was determined for the sand specimens prepared under identical conditions. By comparing the results of these two types of tests, the coefficients for correction were established. The coefficient allowing for the effects of load irregularity in one-direction alone will be denoted by C_2_ and the coefficient, C_5_, will be used to represent the effects of multi-directionality in the seismic loading. Using these two coefficients, the maximum shear stress ratio causing an equivalent level of shear strain of 5% DA, ***τ***_max,_*_ℓ_*/***σ***_o_’ in multi-directional irregular loading can be correlated with the cyclic stress ratio, (***σ***_d_*_ℓ_*/2***σ***_o_’)_20_, inducing the 5% DA strain in 20 cycles of uniform loading in one direction as follows:

[5] $$\frac{\tau_{\text{max},\ell }}{{\sigma_{\text{o}}}^{\prime }}={\text{C}}_{2}\cdot {\text{C}}_{5}{\left(\frac{\sigma_{\text{d }\ell }\;}{2{\sigma_{\text{o}}}^{\prime }}\right)}_{20},$$

where ***τ***_max, _*_ℓ_*denotes the peak shear stress in any irregular time history of shear stress variation which is large enough to induce the 5% DA shear strain.

## Analysis of liquefaction

The analysis of liquefaction can be made by simply comparing the seismically induced shear stress ratio against the similarly expressed shear stress ratio required to cause initial liquefaction or 5% DA shear strain. The externally applied shear stress ratio can be evaluated by Eq. [4] and the corresponding strength expression is given by,

[6] $$\frac{\tau_{\text{max},\ell }}{{\sigma_{\text{v}}}^{\prime }}={\text{C}}_{1}\cdot {\text{C}}_{2}\cdot {\text{C}}_{5}{\left(\frac{\sigma_{\text{d }\ell }\;}{2{\sigma_{\text{o}}}^{\prime }}\right)}_{20},$$

where C_1_ is a coefficient allowing for the effect of K_o_ -condition at the stage of long-term consolidation and given by,

[7] $${\text{C}}_{1}=\frac{1+2{\text{K}}_{\text{o}}}{3}.$$

Note that K_o_ is the ratio, ***σ***_h_’/***σ***_v_’, between the lateral effective stress ***σ***_h_’ and the vertical effective stress ***σ***_v_’ at consolidation and the mean principal stress ***σ***_o_’ is given by ***σ***_o_’ = C_1_***σ***_v_’. It has been known that each of the three coefficients jointly takes a value of C_1_C_2_C_5_ ≅ 1.0.

Using the quantities determined from Eqs. [4] and [6], the liquefaction potential of a sand deposit is evaluated in terms of factor of safety, F*_ℓ_*, which is defined as,

[8] $${\text{F}}_{\ell }=\frac{\tau_{\text{max},\ell }/{\sigma_{\text{v}}}^{\prime }}{\tau_{\text{max}}/{\sigma_{\text{v}}}^{\prime }}.$$

If the factor of safety is equal to or less than unity, liquefaction is said to take place. Otherwise, liquefaction does not occur.

## Recent advances in evaluating cyclic strength

While several factors influencing cyclic strength have been extensively explored in recent years, one of the unknown factors would be the effect of partial saturation. Since the liquefaction is known to occur in sand deposits below the ground water table, a vast majority of the laboratory tests has been conducted on samples of sand which are fully saturated with water. However, the sand layer at shallower depths, say, several metres below the ground water table was found not always fully saturated with water. Thus, the effects of partial saturation have emerged as a new issue of engineering significance to be clarified in order to evaluate the resistance of sand with a greater accuracy.

Laboratory tests have in fact shown that the resistance of sand to the onset of liquefaction tends to increase with a reduction in the saturation ratio S_r_ of soil samples. Since the saturation ratio is a quantity requiring measurement of volume of a soil element, it is generally difficult to determine it precisely particularly when the soil element is partly saturated near the state of full saturation. Then, the B-value has been used instead to quantify the state of saturation. The B-value is defined as the ratio of the induced pore water pressure to the applied total confining stress in undrained conditions, and this value is easily measured in the laboratory tests and accurate enough to indicate a state of partial saturation. Thus, the B-value has been widely used in laboratory soil testing for evaluating the degree of saturation of soil specimens. According to the recent studies by Chaney (1978)5) and Yoshimi *et al*. (1989),6) the resistance to liquefaction has been shown to increase roughly twice as much as that of fully saturated samples, when the B-value drops to a level of about zero with a saturation ratio S_r_ of about 90%.

However, a crucial disadvantage of using either the B-value or saturation ratio S_r_ is that it is practically impossible to monitor these quantities in soil deposits in the field. Then, even if its importance is recognized, there has been no way to monitor the B-value or saturation ratio S_r_ in any method of field investigations and to duly consider its effects under their in-situ conditions in evaluating the liquefaction resistance of in-situ sand deposits.

**Fig. 6:**
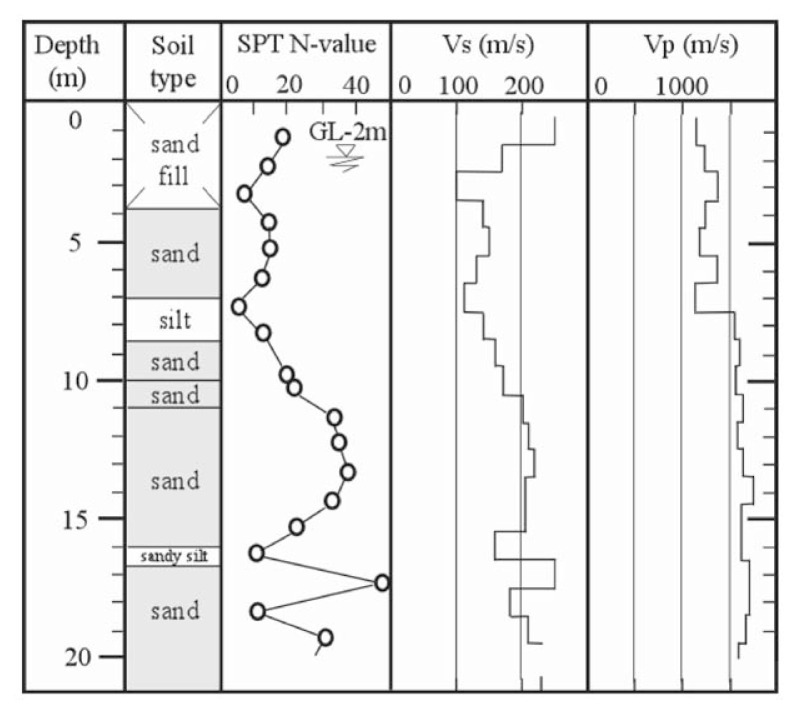
Soil profiles at Shinano estuary in Niigata, Japan.

On the other hand, measurements of propagation velocities of shear wave, V_s_, and longitudinal wave, V_p_, in the field have been carried out at a number of sites by means of the cross-hole and down-hole techniques, which are now in use commonly in routine investigation. With respect to the velocity of the longitudinal wave (P-wave), field measurements have shown that it often yields values which are approximately equal to or somewhat smaller than the P-wave velocity travelling through water even in the case of seemingly saturated loose soil deposits existing at shallow depths below the ground water table. One of such examples of the velocity profile obtained by means of the down-hole method is shown in Fig. 6. This is the soil profile at a site near the mouth of the Shinano River in the city of Niigata, Japan. It may be seen that the velocity of P-wave propagation takes values of V_p_ = 1200~1300 m/sec down to a depth of 7 m. It is generally recognized that it is quite common to observe similar velocity profiles in many other cases. This tendency has also been unearthed by Kokusho (2000)7) in relation to the study of amplification characteristics of longitudinal motions during earthquakes. This fact suggests that the soil deposit several metres below the ground water table is not fully saturated and in a state of near-saturation. Thus, in view of the laboratory-confirmed increased liquefaction resistance of partially but nearly saturated sand as mentioned above, it is highly likely that the in-situ deposits of sands several metres below the ground water table would exhibit resistance to liquefaction which is substantially greater than the value hitherto known and used in the design practice assuming the soils to be fully saturated.

There have been few efforts thus far to measure the P-wave velocity in laboratory soil samples because of lack of recognizing its importance in engineering application. However, the techniques to monitor it in laboratory tests may be explored without much difficulty. Some efforts have been done in this context recently by Nakagawa *et al*. (1996, 1997),8),9) and by Fioravante (2000),10) using a set of bender elements attached to triaxial test specimens. Thus, the use of the P-wave velocity is considered to have a potential as a means to identify the degree of saturation of soils in field deposits as well as in the laboratory. The advantage of using the P-wave velocity as a liaison parameter for identifying the saturation ratio S_r_, and hence the liquefaction resistance may be summarized as follows:

1. P-wave velocity can be measured both in the field deposits and in laboratory soil samples while other index properties such as B-value and saturation ratio S_r_ can by no means be monitored in the field. Therefore, the P-wave velocity may be used as a common parameter to identify conditions of laboratory soil samples and that in-situ soil deposits as well in relation to the degree of saturation.
2. P-wave velocity measured in-situ is considered to possess an equal level of credibility to that monitored in the laboratory, and therefore it could be used to identify the in-situ B-value as it is used for the laboratory soil samples.
3. The measurements of P-wave do not induce any disturbance to intact soils both in the field and in the laboratory, because the shear strain induced is infinitesimally small. It is considered as a nondestructive test. Thus, the cyclic tests performed after the P-wave measurements are completely free from effects of sample disturbance.

On the basis of the conceptions as above, multiple series of laboratory tests were conducted on reconstituted samples of sand with varying saturation ratios. The outcome of these tests is described in the following pages of this paper, along with some theoretical consideration to support interpretation of test data based on the theory of elastic porous medium.

## Relations between P-wave, S-wave velocity and B-value

An attempt to correlate the B-value with the P- and S-wave velocities has been made by Kokusho (2000)7) based on the theory of wave propagation through a poroelastic medium. The following is an alternative approach by Ishihara (1996)11) leading to the same formulae.

**Fig. 7:**
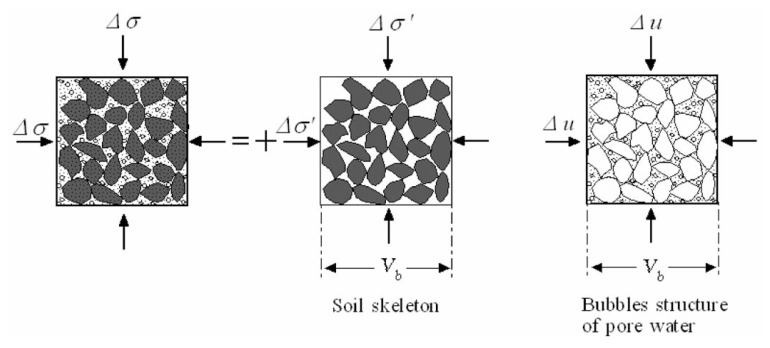
Partition of the effective stress **Δ*σ***’ and pore water pressure **Δ**u.

Let it be assumed that the partially saturated soil is composed of the skeleton and the pores containing air as illustrated in Fig. 7. It is assumed that the bubble-like structure of the pores is much more compressible than water itself because of the air dispersed in the water. It is also assumed that the volume of air is small enough so as not to develop any interaction with the soil skeleton such as meniscus where the degree of saturation is probably below the level of S_r_ = 90%. Let an overall compressional stress, **Δ*σ***, be applied to an element of the partly saturated soil. The stress is divided into two parts; the effective stress, **Δ*σ***’, transmitted to the soil skeleton and the pore water pressure, **Δ***u*, carried by the air-containing water, as schematically illustrated in Fig. 7. Thus, one obtains,

[9] $$\Delta \sigma =\Delta \sigma^{\prime }+\Delta u.$$

Let it be assumed first that the soil skeleton and the bubble structure of pore fluid are deforming independently without mutual interaction. If the volume of the soil skeleton V_b_ is compressed by an amount **Δ**V_b_ due to an increase in effective stress **Δ*σ***’, the following relation is obtained,

[10] $$\frac{\Delta {\text{V}}_{\text{b}}}{{\text{V}}_{\text{b}}}={\text{C}}_{\text{b}}\;\Delta \sigma^{\prime },$$

where C_b_ is the compressibility of the soil skeleton. By denoting the porosity with n, the volume of the void consisting of water plus air is given by nV_b_. Then, if the pore air-water is compressed by an amount **Δ**V_w_ due to the pore pressure increase **Δ**u, the following relation is obtained,

[11] $$\frac{\Delta {\text{V}}_{\text{w}}}{{\text{nV}}_{\text{b}}}={\text{C}}_{\ell }\Delta \text{u},$$

where C*_ℓ_*is the compressibility of the air-bearing water which is apparently larger than the compressibility of water itself. At this stage, let it be assumed that some constraints are imposed on the occurrence of **Δ**V_b_ and **Δ**V_w_, in such a way that the volume change in each of the two phases cannot occur independently. These constraints are associated with the drainage conditions of pore-filling air-water. If the amount **Δ**V_w_ is greater than **Δ**V_b_, water is entering into the pores from outside, and conversely if **Δ**V_w_ < **Δ**V_b_, water is expelled out of the pores. These two situations imply the conditions where drainage is taking place. If **Δ**V_w_ = **Δ**V_b_, there is no water coming in and out of the pores. Thus, it is obvious that the undrained condition is imposed by,

[12] $$\Delta {\text{V}}_{\text{b}}=\Delta {\text{V}}_{\text{w}}.$$

This is regarded as a kind of a compatibility condition or a constraint required for otherwise independently deforming two-phase media. In fact, the condition of Eq. [12] requires that the soil skeleton and the pore bubble structure should deform together by the same amount. Introducing Eqs. [10] and [11] into the undrained condition of Eq. [12], and using the relation of Eq. [9], one obtains,

[13] $$\frac{\Delta {\text{V}}_{\text{b}}}{{\text{V}}_{\text{b}}}=\frac{{\text{nC}}_{\ell }}{1+\frac{{\text{nC}}_{\ell }}{{\text{C}}_{\text{b}}}}\Delta \sigma .$$

If the soil skeleton is viewed as an elastically deforming medium, then the overall volumetric modulus, K, is defined from Eq. [13] as,

[14] $$\text{K}=\frac{1+\frac{{\text{nC}}_{\ell }}{{\text{C}}_{\text{b}}}}{{\text{nC}}_{\ell }}.$$

It is well known that the pore pressure coefficient, B, as explained above is given as the ratio of **Δ**u/**Δ*σ*** and defined with reference to Eqs. [9], [10] and [11], as follows:

[15] $$\text{B}=\frac{1}{1+\frac{{\text{nC}}_{\ell }}{{\text{C}}_{\text{b}}}},\;\;\;\text{or }\;\;{\text{C}}_{\ell }=\frac{1-\text{B}}{\text{B}}\frac{{\text{C}}_{\text{b}}}{\text{n}}.$$

Eq. [15] is interpreted as a relation of the B-value which is expressed in terms of the compressibility of the aircontaining pore water, C*_ℓ_*, and the compressibility of the skeleton, C_b_.

In the theory of elasticity, it is known that Poisson’s ratio, ***ν***, which will be referred to as overall Poisson’s ratio, is expressed as a function of the shear modulus G_o_ and the volumetric modulus K as follows:

[16] $$\nu =\frac{1}{2}\frac{3\text{K}-2{\text{G}}_{\text{o}}}{3\text{K}+{\text{G}}_{\text{o}}}.$$

Note that this equation refers to the parameters ***ν***, K and G_o_, which are defined for overall deformation, not for the soil skeleton deformation. The modulus G_o_ entering in Eq. [16] is the elastic shear modulus of the skeleton at small strains which is also equal to the shear modulus defined for overall deformation. Note that in the theory of poro-elasticity the resistance to shear is mobilized only by skeleton and consequently the shear modulus for overall deformation is considered identical to that of the skeleton deformation. Introducing Eq. [15] into Eq. [14], one obtains,

[17] $$\text{K}=\frac{1}{{\text{nC}}_{\ell }\text{B}}=\frac{1}{(1-\text{B}){\text{C}}_{\text{b}}}.$$

It is thus known that the volumetric modulus of overall deformation, K, is related to the compressibility of the soil skeleton defined as, K_b_ = 1/C_b_, and the B-value. With reference to Eq. [17], Eq. [16] is rewritten as,

[18] $$\nu =\frac{1}{2}\frac{3-2{\text{G}}_{\text{o}}{\text{nC}}_{\ell }\text{B}}{3+{\text{G}}_{\text{o}}{\text{nC}}_{\ell }\text{B}}=\frac{1}{2}\frac{3-2{\text{G}}_{\text{o}}{\text{C}}_{\text{b}}(1-\text{B})}{3+{\text{G}}_{\text{o}}{\text{C}}_{\text{b}}(1-\text{B})}.$$

It is to be noted that the overall Poisson’s ratio, ***ν***, for the overall deformation is expressed in terms of the soil skeleton compressibility C_b_, shear modulus of the soil skeleton G_o_ and the B-value. On the other hand, according to the theory of elastic wave propagation, the overall Poisson’s ratio ***ν***, velocity of S-wave propagation, V_s_, and P-wave propagation, V_p_, is related as follows:

[19] $${\left(\frac{{\text{V}}_{\text{p}}}{{\text{V}}_{\text{s}}}\right)}^{2}=\frac{2(1-\nu )}{1-2\nu }.$$

Introducing Eq. [18] into Eq. [19], one obtains,

[20] $${\left(\frac{{\text{V}}_{\text{p}}}{{\text{V}}_{\text{s}}}\right)}^{2}=\frac{4}{3}+\frac{1}{{\text{G}}_{\text{o}}{\text{G}}_{\text{b}}(1-\text{B})}.$$

It is to be noted here that the quantity G_o_ and C_b_ are the parameters related to the soil skeleton deformation without any influence of pore water. Thus, the relation of Eq. [20] implies that the ratio of V_p_ and V_s_, pertaining obviously to the overall gross quantities is correlated with the soil skeleton parameter G_o_C_b_ and the pore pressure coefficient B, if the undrained condition as expressed by Eq. [12] is assumed to hold valid. Since the wave propagation through near-saturated soils takes place in a sufficiently short period of time without allowing any drainage of pore water, the correlation of Eq. [12] is considered to hold valid.

To understand the above concept more visibly, it might be more convenient to express G_o_C_b_ in terms of Poisson’s ratio of the soil skeleton, ***ν***_b_, which may be defined as follows, in a manner similar to the definition of Eq. [16]:

[21] $$\nu_{\text{b}}=\frac{1}{2}\frac{3{\text{K}}_{\text{b}}-2{\text{G}}_{\text{o}}}{3{\text{K}}_{\text{b}}+{\text{G}}_{\text{o}}}=\frac{1}{2}\frac{3-2{\text{G}}_{\text{o}}{\text{C}}_{\text{b}}}{3+{\text{G}}_{\text{o}}{\text{C}}_{\text{b}}},\;\;\;{\text{C}}_{\text{b}}=\frac{1}{{\text{K}}_{\text{b}}},$$

where the soil skeleton modulus K_b_ is converted to the compressibility of the soil skeleton C_b_ through the relation C_b_ = 1/K_b_. By introducing the G_o_C_b_ from Eq. [21] into Eq. [20], one obtains,

[22] $${\left(\frac{{\text{V}}_{\text{p}}}{{\text{V}}_{\text{s}}}\right)}^{2}=\frac{4}{3}+\frac{2(1+\nu_{\text{b}})}{3(1-2\nu_{\text{b}})(1-\text{B})}.$$

This is the relation expressing the ratio V_p_/V_s_ in terms of the skeleton Poisson’s ratio ***ν***_b_ and the B-value. Eq. [22] is the same formula as that derived by Kokusho (2000).7) In the studies described below, the deformation of the soil induced by the wave propagation is considered very small, thereby keeping the strain level within an elastic range. Therefore, the value of ***ν***_b_ is assumed to take a constant value irrespective of the B-value. It is also possible to derive the relation between the skeleton Poisson’s ratio ***ν***_b_ and overall Poisson’s ratio ***ν***. This can be done by introducing G_o_C_b_ from Eq. [21] into Eq. [18] as follows:

[23] $$\nu =\frac{3\nu_{\text{b}}+(1-2\nu_{\text{b}})\text{B}}{3-(1-2\nu_{\text{b}})\text{B}}.$$

This relation was also derived by Kokusho (2000).7) In Eq. [23], it is known that ***ν***= ***ν***_b_ when B = 0. This implies that ***ν***_b_ is the Poisson’s ratio when there is no pore pressure buildup. Thus, the value of ***ν***_b_ is to be taken as the Poisson’s ratio at a partly saturated state with B = 0 where the corresponding saturation ratio drops to a value of about S_r_ ≅ 90%.

## Laboratory tests

**Fig. 8:**
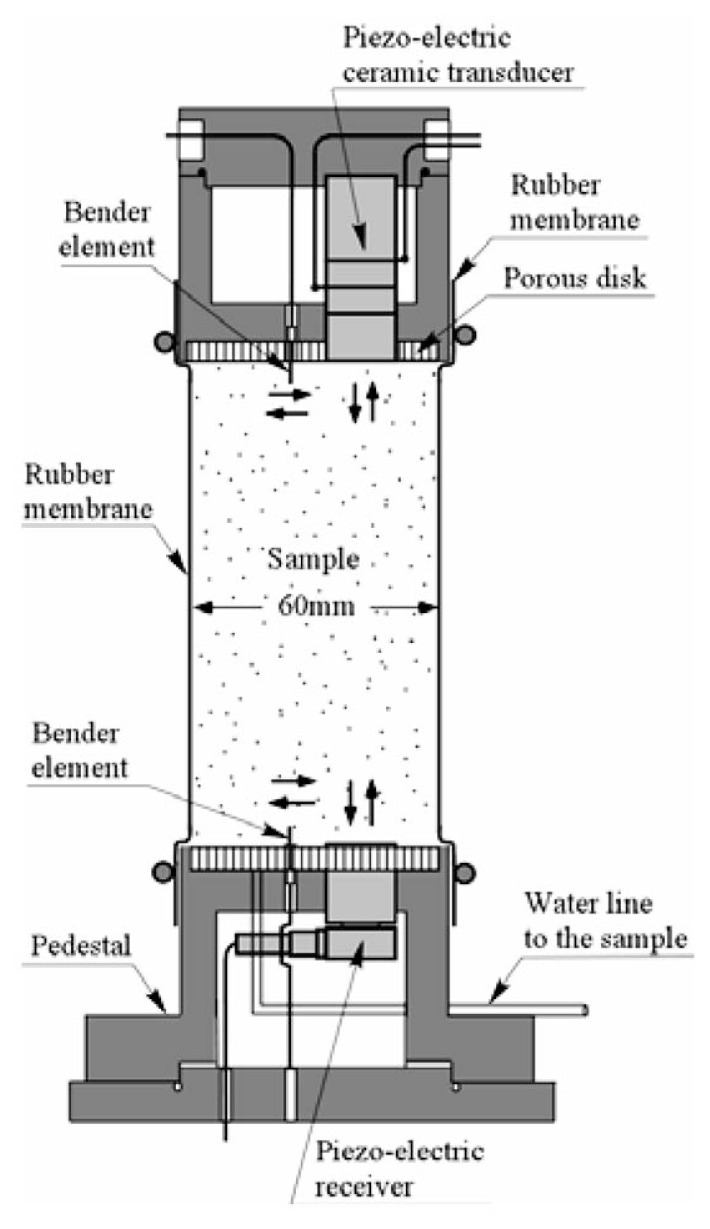
Cross sections of the cap, sample and pedestal with transducers in the triaxial cell.

The cylindrical cell as shown in Fig. 8 which accommodates a triaxial specimen of 60 mm in diameter and 120 mm in height, was newly fabricated for this study (Tsukamoto *et al*. 2002).12) Located at the top end of a soil specimen, there is a cap equipped with a porous disk, which houses a triggering system of the longitudinal and shear wave pulses. At the bottom end of the soil specimen there is a pedestal, also equipped with a porous disk, and houses receivers of the longitudinal and shear wave pulses generated at the cap and travelling through the soil specimen.

The transducer for triggering the impulse of the P-wave adopted in this study is a piezo-electrically driven bolt-clamped ceramic transducer, which is about 15 mm in diameter and 42 mm in length. This transducer is capable of generating impulsive excitation of up to 60 kHz to trigger P-waves whose direction of motion is in parallel to the direction of wave propagation. However, only a single impulse excitation was employed in this study. It is difficult, therefore, to identify exact values of frequency at which the tests were performed. The transducer for receiving the P-wave is a piezo-electric accelerometer, which is about 17.5 mm in diameter and 9.8 mm in length. This transducer is mechanically fixed to the base of a dummy dualuminium-made block. This dummy block is then plugged into a pedestal of a diameter of 65 mm, and comes in contact with the triaxial specimens. A pair of bender elements were used to trigger and to receive the shear wave impulse which travels through the triaxial specimen. The bender element is a piezo-electrically driven rectangular ceramic tip, which can be excited by applying voltage input and gain voltage output. The end part of each bender element is plugged into the cap and pedestal. The voltage input is applied to the bender element at the top cap, which moves back and forth and causes the shear wave whose direction of motion is perpendicular to the direction of wave propagation. The bender element fixed to the pedestal is excited upon arrival of the shear wave. The velocity of the Pas well as S-wave can be calculated by measuring the time difference between excitation and arrival.

**Fig. 9:**
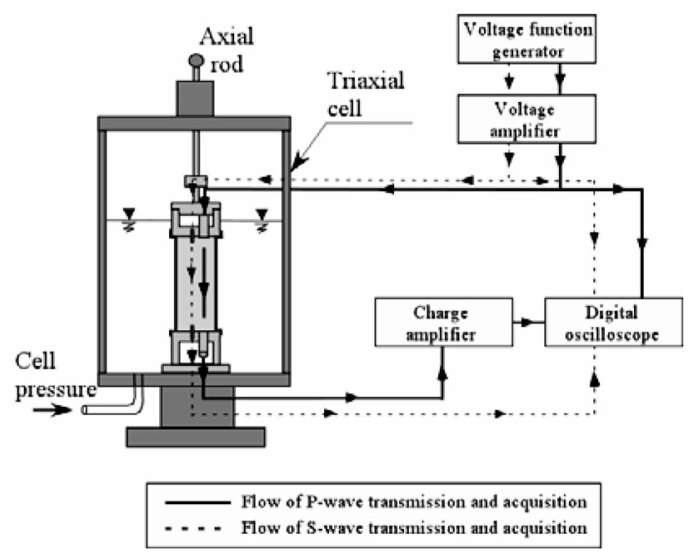
Flow of signal transmission and acquisition for wave velocity measurement in the triaxial test.

The layout of the triaxial test apparatus and monitoring system is shown in Fig. 9. Soil specimens 60 mm in diameter and 120 mm in height were prepared by the method of air pluviation in the rubber membrane to achieve the relative density of D_r_ = 40 and 60%. The sample was put into the triaxial cell and tested as shown in Fig. 9. Note that the pore water within the sample is completely sealed and separated from the water in the triaxial cell. The soil specimens were isotropically consolidated to a confining stress ***σ***_o_’ = 98 kPa, while allowing the pore water to go out. Then after closing the valve of water line leading to the inside of the soil sample, the B-value was measured. It is known that the B-value increases with increasing back pressure, which is defined as the pressure applied to the pore water itself. The back pressure can be adjusted easily by increasing or decreasing the cell pressure simultaneously with the pressure leading to the sample enclosed by the rubber membrane. After measuring the B-value, the measurements of the velocities of the P- and S-wave propagation were carried out. After finishing non-destructive wave velocity measurements, the cyclic loading triaxial tests were conducted with a given cyclic stress ratio, ***σ***_d_/(2***σ***_o_’), by employing a sinusoidal cycles of 0.1 Hz, where ***σ***_d_ is the amplitude of the cyclic axial stress, and ***σ***_o_’ is the effective confining stress at the time of consolidation.

## Results of tests on P- and S-wave measurements

**Fig. 10:**
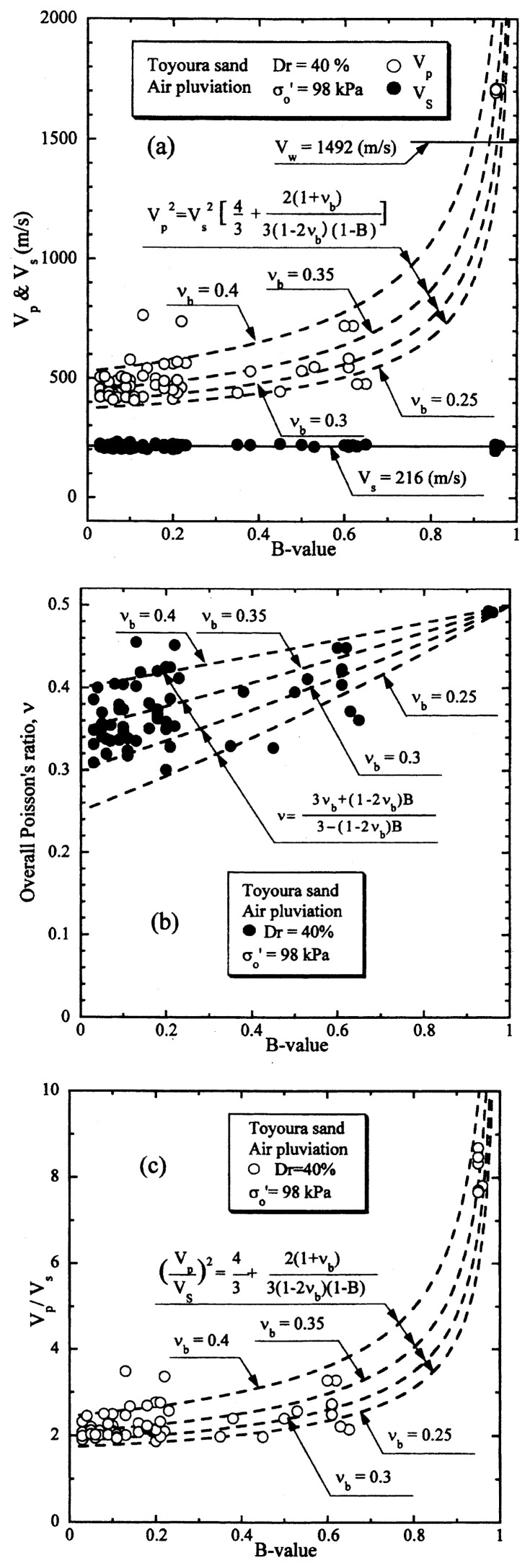
P-wave and S-wave velocities versus B-value (Dr = 40%).

The results of the P- and S-wave measurements on samples of Toyoura sand prepared to a relative density of D_r_ = 40% are displayed in Fig. 10(a) in terms of V_s_ and V_p_ values plotted versus the B-value. The outcome of the tests shown in Fig. 10(a) indicates that the velocity of S-wave propagation takes on a value of V_s_ = 216 m/sec and remains unchanged with an increase in the B-value. It is to be noted that the smallest B-value was actually B = 0.05 where the corresponding saturation ratio was about S_r_ = 90%, and the largest B-value achieved in the tests was B = 0.95 with S_r_ = 100%.

The velocity of propagation of P-wave is also shown in Fig. 10(a) where it is seen that the value of V_p_ tends to increase significantly with an increase in the B-value from a value of about V_p_ = 500 m/sec at B = 0 to V_p_ = 1700 m/sec at B = 0.95. Shown also in Fig. 10(a) is the propagation velocity of compressional wave through water V_w_ = 1492 m/sec that is achieved at the temperature of 20 ˚C. In the case of the P-wave propagation through fully saturated soils, the stiffness of soils comes into effect to some extent because of lateral constraint being imposed on the deformation of the soils during the wave propagation. Therefore, the P-wave through fully saturated soils was shown to propagate with a velocity which is 10–15% faster than the velocity through water, (Ishihara 1971).13) The result of P-wave measurement on the sand as above at full saturation with B = 0.95 is coincident with the outcome of the earlier study.

With reference to the relation of Eq. [20], the P-wave velocity of propagation is known to be expressed as a function of the shear modulus G_o_ and the compressibility of the soil skeleton C_b_. As C_b_ is simply a reciprocal of the volumetric modulus K_b_, Eq. [20] implies the fact that the ratio of the P-wave and S-wave propagation velocity is expressed as a function of the ratio of volumetric and shear modulus of the soil skeleton, namely, G_o_/K_b_, and the B-value. It is to be remembered that the ratio G_o_/K_b_ reflects the deformation characteristics of the soil skeleton at small strains in an elastic range. Thus, it was considered to be more physically visible, if the ratio G_o_/K_b_ is expressed in terms of the skeleton Poisson’s ratio ***ν***_b_ which can be defined by Eq. [21]. Thus, with the use of ***ν***_b_, the relation of Eq. [20] was converted to Eq. [22].

To provide a physical basis for interpreting the results of the tests, the values of V_p_ computed by Eq. [22] are shown superimposed in Fig. 10(a) versus the B-value for various postulated values of the skeleton Poisson’s ratio. Looking over the data points, it would be reasonable to assume that the skeleton Poisson’s ratio would be approximately ***ν***_b_ = 0.35.

Based on the measured values of V_p_ and V_s_, corresponding to small strain elastic range, the overall Poisson’s ratios, ***ν***, were determined through Eq. [19] and these values are shown in Fig. 10(b) in terms of the plot versus the measured B-value. Also shown superimposed is a set of theoretical curves from Eq. [23] for various values of ***ν***_b_. The comparison of the test data with those obtained from Eq. [23] indicates that the dependency of the skeleton Poisson’s ratio ***ν***_b_ on the B-value is not necessarily clear. However, the value of ***ν***_b_ = 0.35 would be taken as a reasonable average value representing the overall trend of variation of ***ν*** with the B-value.

The test data on the P- and S-wave propagation shown in Fig. 10(a) are alternatively displayed in Fig. 10(c), where the ratio V_p_/V_s_ for each of test data is plotted versus the B-value. The plot in this format would be more reasonable in the sense that it can provide a comparison with the theoretically derived curves. Thus, the relation of Eq. [22] is also shown plotted in Fig. 10(c) for comparison sake. It may be seen as well that the value of ***ν***_b_ = 0.35 could represent, with a reasonable degree of accuracy, the overall feature of variation of V_p_/V_s_ with the B-value.

**Fig. 11:**
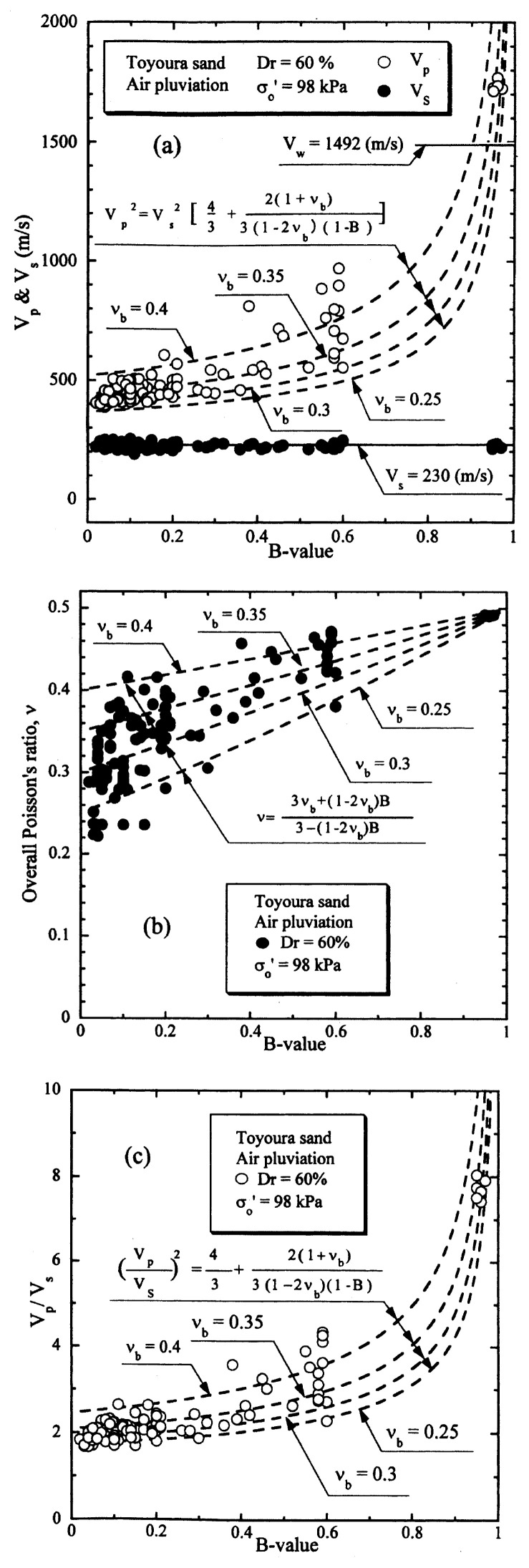
P-wave and S-wave velocities versus B-value (Dr = 60%).

A similar set of test data on Toyoura sand for the relative density of D_r_ = 60% under otherwise identical conditions is presented in Fig. 11. Looking over the whole sets of test data shown in Figs. 10 and 11 in the light of theoretical considerations, it may be concluded that the relation between V_p_ or V_p_/V_s_ and B-value for Toyoura sand can be represented to a satisfactory level of coincidence by the theoretical relation of Eq. [22], irrespective of the relative density, if the skeleton Poisson’s ratio is chosen as ***ν***_b_ = 0.35. It is of interest to note that the skeleton Poisson’s ratio ***ν***_b_ remains almost the same independent of the relative density. Other series of similar tests performed under varying confining stresses have shown that the skeleton Poisson’s ratio also remains the same irrespective of the confining stress at the time of the consolidation.

## Results of cyclic loading tests

**Fig. 12:**
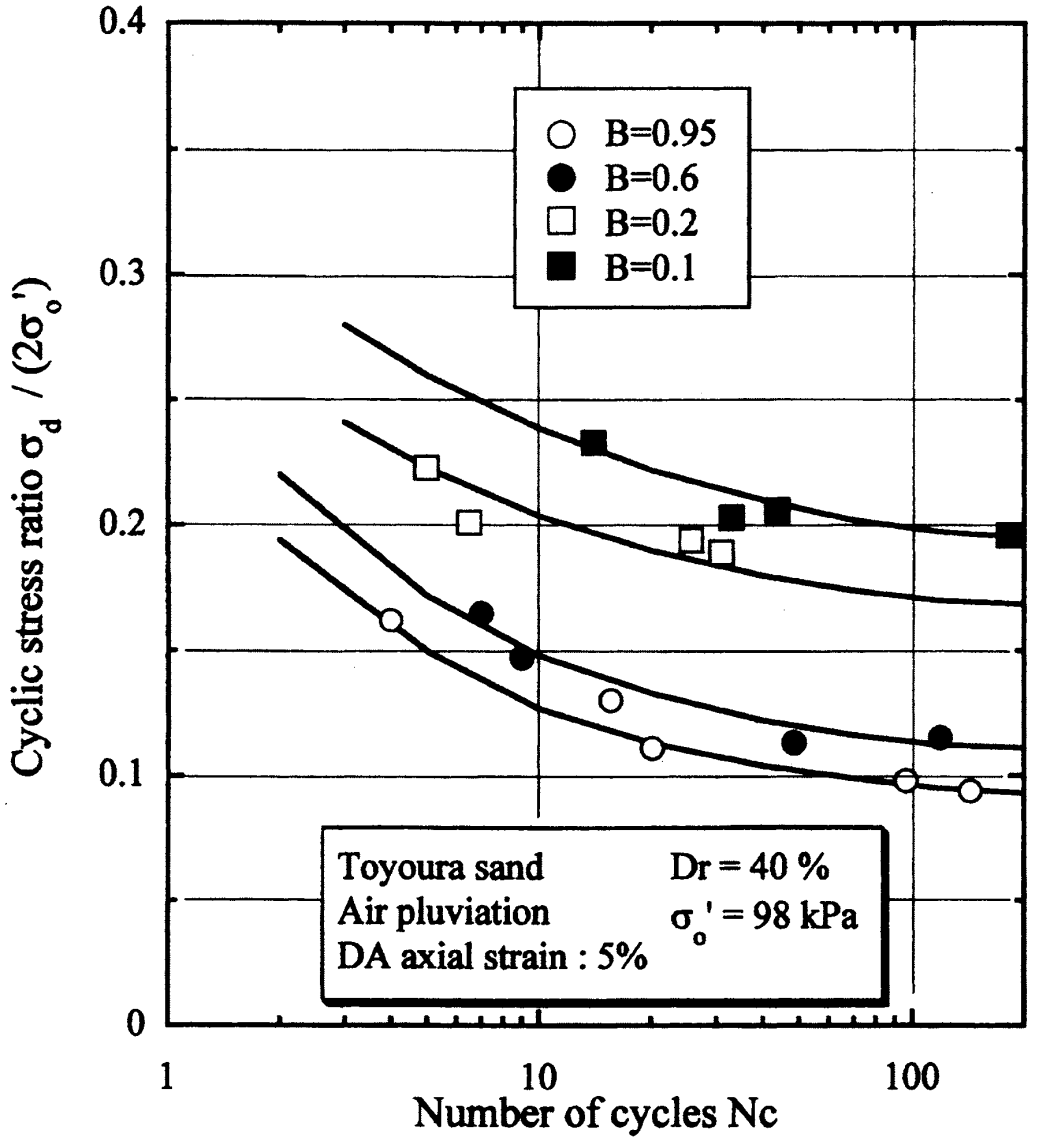
Cyclic stress ratio versus number of cycles causing 5% DA axial strain (Dr = 40%).

**Fig. 13:**
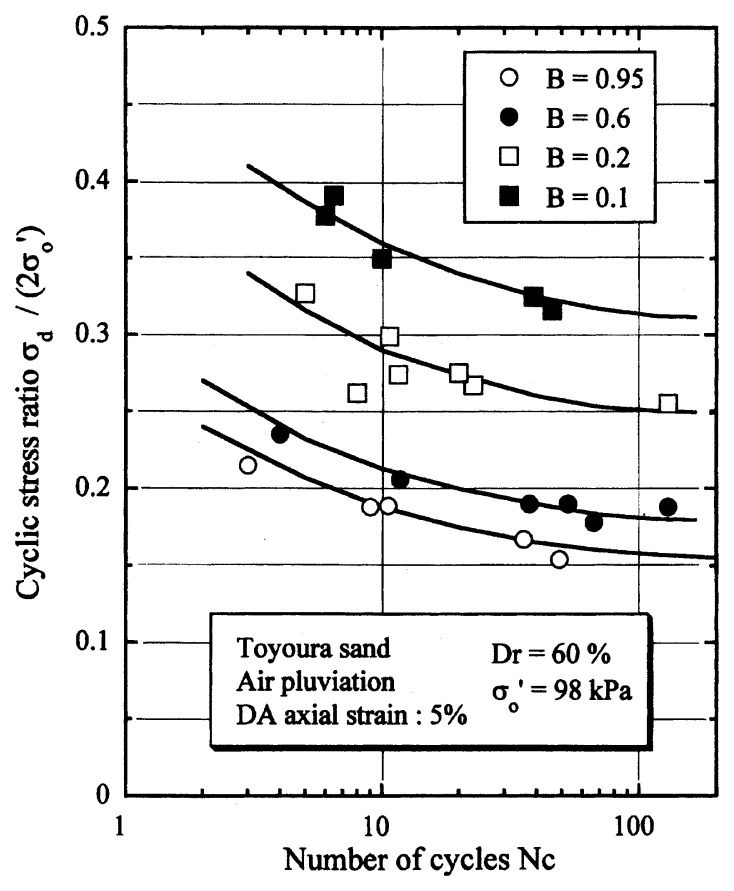
Cyclic stress ratio versus number of cycles causing 5% DA axial strain (Dr = 60%).

**Fig. 14:**
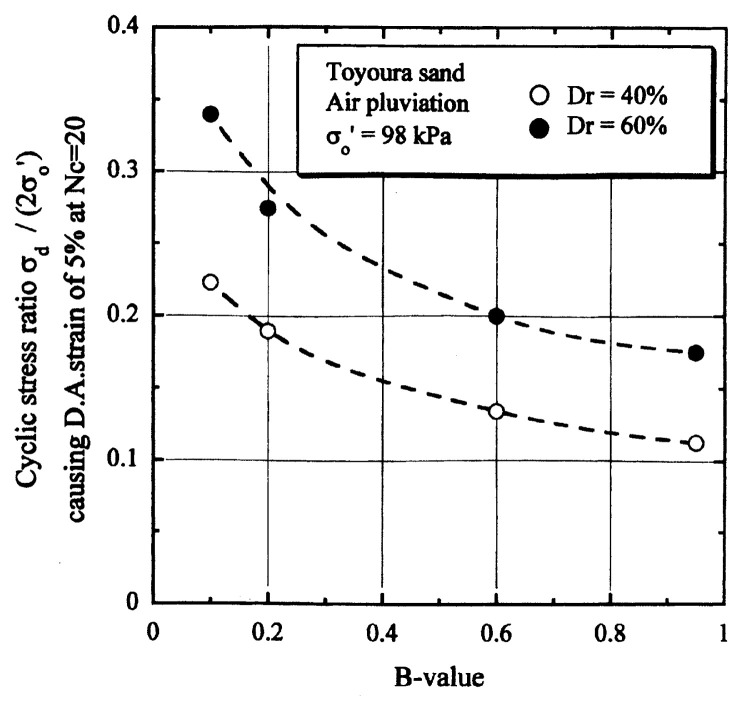
Cyclic strength versus B-value.

**Fig. 15:**
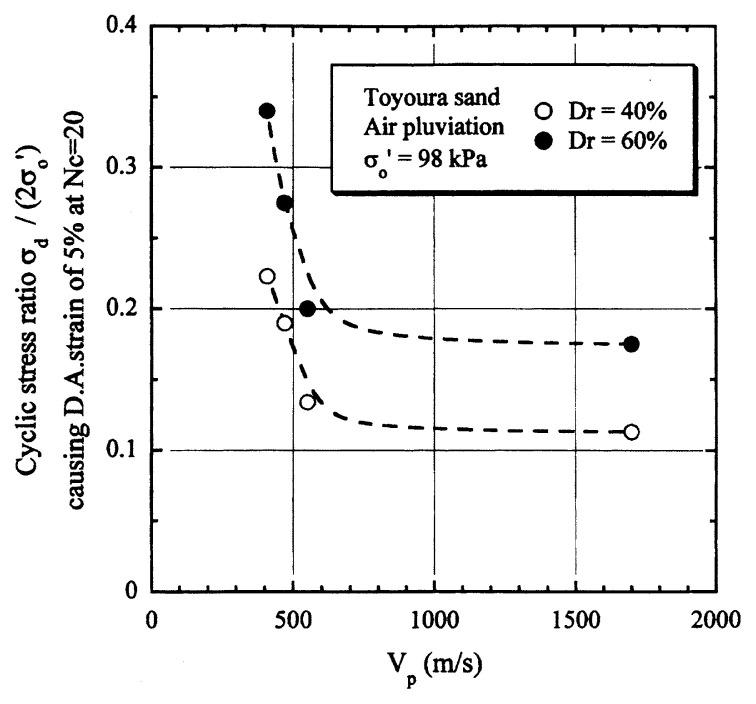
Cyclic strength versus P-wave velocity V_p_.

**Fig. 16:**
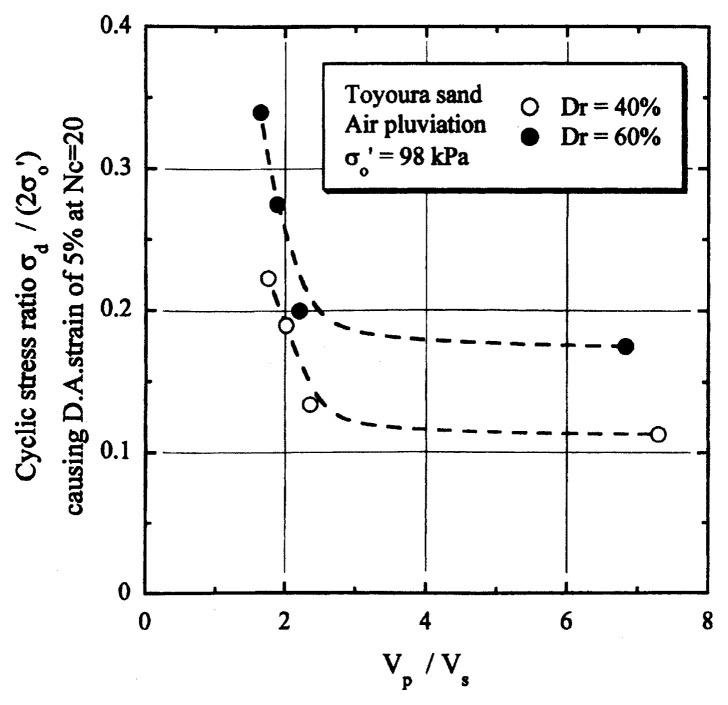
Cyclic strength versus V_p_/V_s_.

**Fig. 17:**
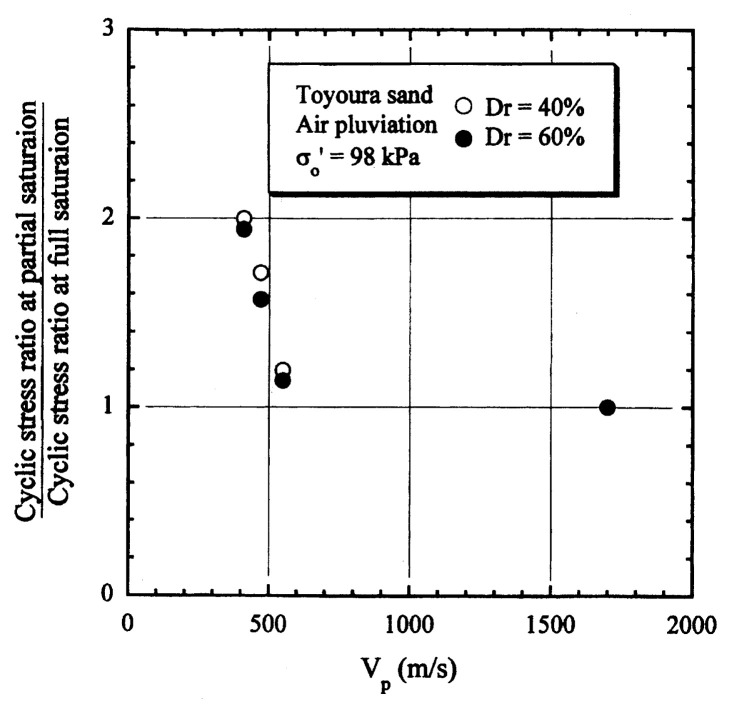
Normalized cyclic strength versus P-wave velocity V_p_.

**Fig. 18:**
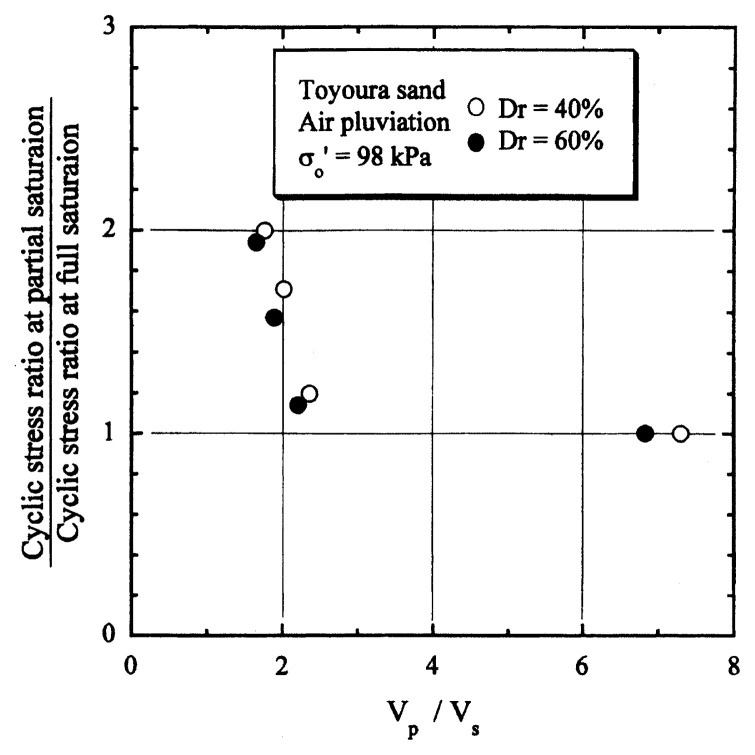
Normalized cyclic strength versus the ratio of velocities V_p_/V_s_.

After performing measurements of P-wave and S-wave velocities, the samples with different B-values were subjected to a series of cyclic axial stresses with constant amplitudes under undrained conditions until they deformed to the double-amplitude (DA) axial strain of 5%. The amplitude of the cyclic axial stress, ***σ***_d_, divided by twice the confining stress, 2***σ***_o_’, which was necessary to induce 5% DA axial strain is plotted in Figs. 12 and 13 versus the number of cycles, N_c_, for the case of the samples prepared at a relative density of D_r_ = 40% and 60%, respectively. It becomes obvious from these figures that the cyclic stress ratio needed to induce 5% DA axial strain tends to increase significantly with decreasing degree of saturation as represented by the B-value. The cyclic stress ratio inducing the 5% DA axial strain in 20 cycles of load application is read off from Figs. 12 and 13 and plotted versus the B-value in Fig. 14. It is apparent in these figures that the cyclic strength as defined above tends to increase with decreasing B-value, and hence with decreasing saturation ratio S_r_ to a value of about 90%. The similarly defined cyclic strength is presented in Fig. 15 as a function of the P-wave velocity V_p_ measured at the start of each of the cyclic loading tests, where it is seen that the cyclic resistance tends to increase significantly with a decrease in V_p_ particularly when the P-wave velocity becomes less than 500 m/sec and where the B-value drops to less than 0.1 with the saturation ratio S_r_ of 90%. The same data set is alternatively shown in Fig. 16 as a function of V_p_/V_s_ where it is seen that the cyclic strength begins to increase sharply when the velocity ratio V_p_/V_s_ drops to a value of about 3. The cyclic strength defined above was normalized to a value at full saturation with the B-value of 0.95. The normalized cyclic strength is displayed in Figs. 17 and 18 as a function of V_p_ and V_p_/V_s_, respectively. It may be seen in these figures that the cyclic strength becomes larger as the V_p_ or V_p_/V_s_ decreases and could take a value about twice as much as the cyclic strength at full saturation, when the V_p_-value drops to 400 m/sec, or when the velocity ratio V_p_/V_s_ becomes equal to about 1.8. Note that at the state of V_p_/V_s_ = 1.8, the B-value is practically equal to zero, but the saturation ratio still remains as high as about S_r_ = 90%.

**Fig. 19:**
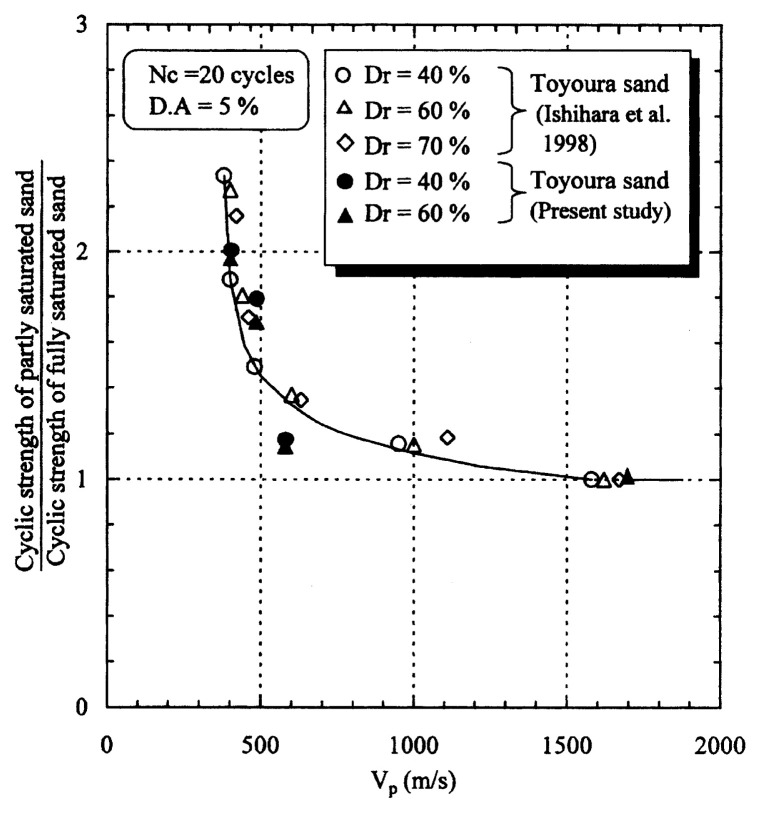
Summary plots of the test data on the normalized cyclic strength of sands.

In order to examine the general tendency, the data of similar tests performed previously on Toyoura sand, (Ishihara *et al*. 1998),14) were collected and shown in Fig. 19 together with the data from the present study. Note that the test data in the previous studies were obtained using another test apparatus. The summary plot shown in Fig. 19 indicates that the cyclic strength of partly saturated Toyoura sand tends to increase with decreasing B-value, and that the cyclic strength reaches a value twice as much as that at full saturation when the B-value drops to zero with the saturation ratio S_r_ of about 90%.

## Case studies

In an effort to apply the above methodology for assessing liquefaction potential of sandy deposits, two sites were chosen and investigated in detail by carrying out in-situ boring, penetration tests, and by recovering undisturbed samples and testing them in the laboratory. The triaxial tests as mentioned in the foregoing sections were then performed and finally assessment of liquefaction potential was made for these two sites. The details of this study are described in a paper by Nakazawa *et al*. (2004).15)

### 1. Koshigaya site

**Fig. 20:**
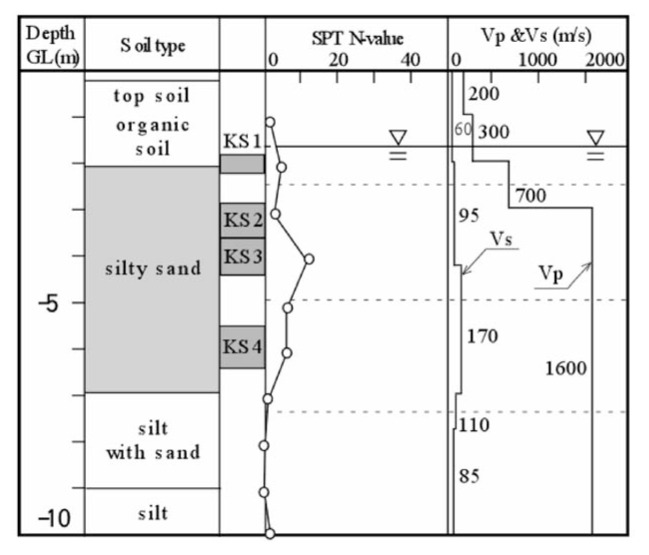
Soil profiles and field test results at lowland area in Koshigaya, Saitama, Japan.

This site is located at Saitama Prefecture in the northern suburb of metropolitan Tokyo, and corresponds to the forefront of the boundary between densely packed residential districts of metropolitan Tokyo and areas yet to be developed. It is known that soft soil deposits prevail widely over this low-land area, which is easily flooded during heavy rainfall. The development of residential districts has recently been planned around this area, and the location of field surveys for the present study was chosen at a site in such areas. At this site, a set of field surveys was conducted, including Standard Penetration Test (SPT), down-hole velocity logging and undisturbed soil sampling. The results are shown in Fig. 20. The undisturbed soil sampling was separately conducted at the location adjacent to the borehole. The symbols of “KS1” to “KS4” in Fig. 20 indicate the depths of undisturbed soil sampling. It is seen in Fig. 20 that the layer of organic soils about 2 m thick lies near the ground surface, under which the layer of silty sand exists. The groundwater table is located at a depth of 1.7 m. The velocity of P-wave propagation was found to be V_p_ = 700 m/s below the groundwater table, but immediately increases to V_p_ = 1600 m/s. The undisturbed soil sampling was carried out at several depths within the silty sand layer.

### 2. Takenouchi site

**Fig. 21:**
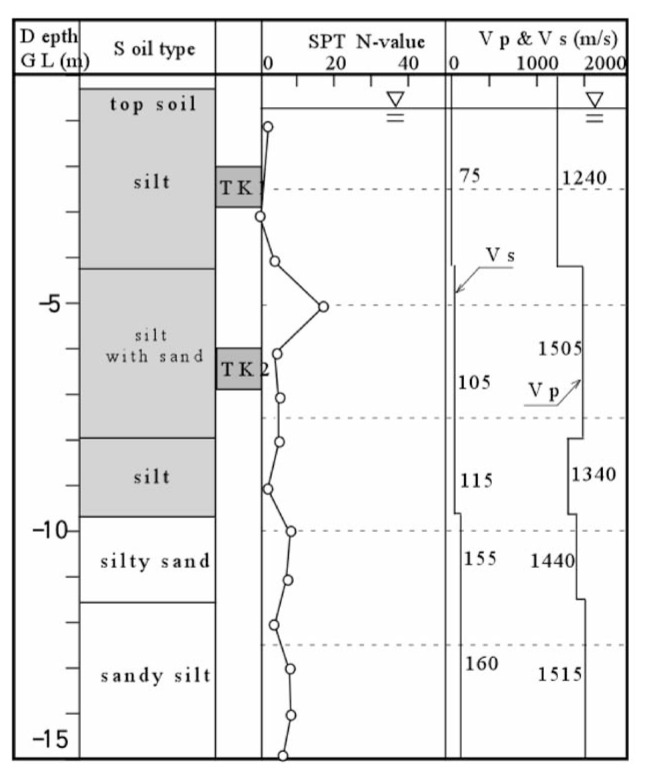
Soil profiles and field test results at Takenouchi industrial village in Sakaiminato, Tottori, Japan.

This site is located in the reclaimed area in Takenouchi industrial district in the city of Sakai-minato, Tottori Prefecture. During Tottoriken Seibu Earthquake on October 6, 2000, the reclaimed deposits in this area extensively liquefied and the surface of almost entire area was innundated by erupted subsurface silt. The same set of field surveys as above was conducted and the results are displayed in Fig. 21. The undisturbed soil sampling was separately conducted at the location adjacent to the borehole. The depth of the undisturbed soil sampling is indicated by “TK1” and “TK2” in Fig. 21 where it is seen that a reclaimed deposit of silt exists down to a depth about 10 m. The groundwater level is located about 1.5 m below the ground surface. The velocity of P-wave propagation was found to be V_p_ = 1240 m/s to a depth of about 3 m below the groundwater table, and increases to V_p_ = 1400 to 1500 m/s.

## Results of laboratory tests on undisturbed samples

**Fig. 22:**
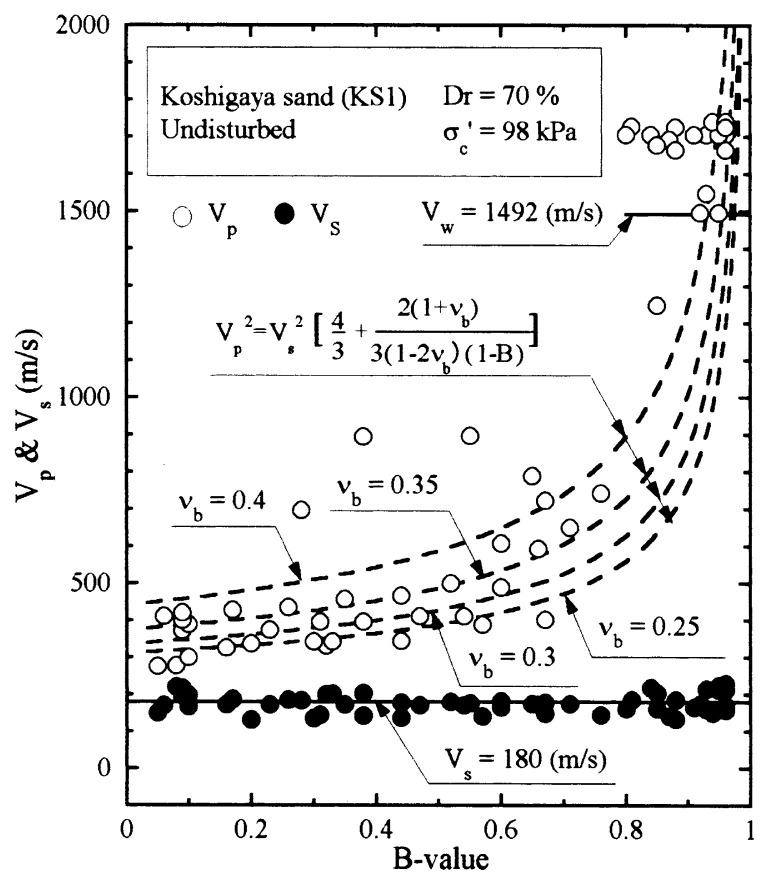
Plots of P-wave and S-wave velocities against B-value measured in laboratory tests (Koshigaya sand, Dr = 70%).

The results of P-wave and S-wave velocity measurements on the undisturbed samples of Koshigaya sand are shown in Fig. 22. The measured values of V_p_ and V_s_ are plotted against the B-value. It is seen that the velocity of S-wave propagation stays constant at V_s_ = 180 m/s independent of the B-value. On the other hand, the velocity of P-wave propagation is seen varying with B-values. To compare the test data with the theoretically derived relationship of Eq. [22], the reference curves are drawn assuming various values of ***ν***_b_ = 0.25, 0.3, 0.35 and 0.4 with the value of V_s_ = 180 m/s. Looking over the data points, it would be reasonable to assume that the skeleton Poisson’s ratio is about ***ν***_b_ = 0.35 for Koshigaya sand.

**Fig. 23:**
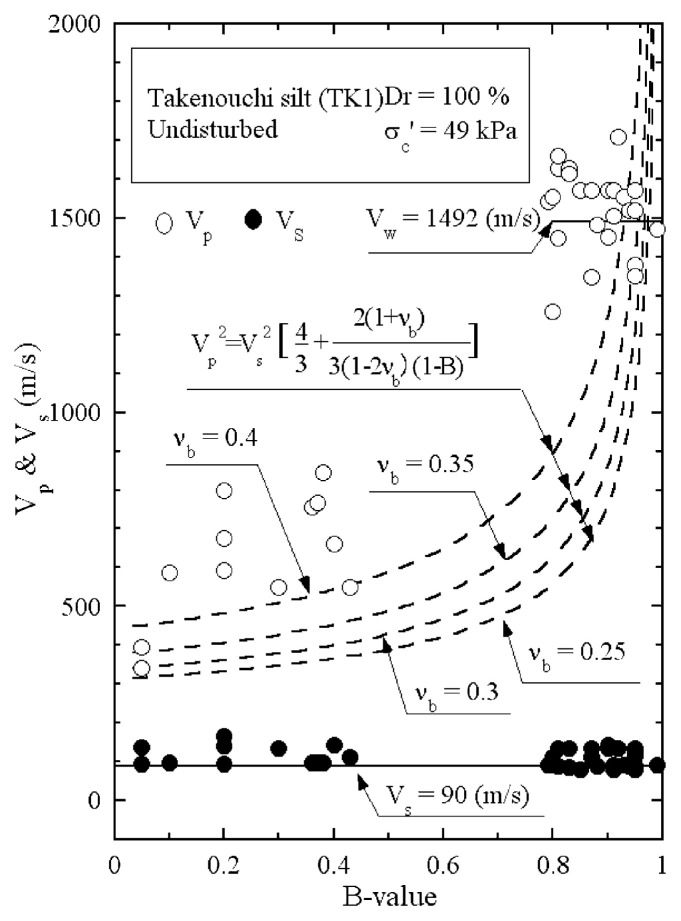
Plots of P-wave and S-wave velocities against B-value measured in laboratory tests (Takenouchi silt, Dr = 100%).

The results of P-wave and S-wave velocity measurements on the undisturbed samples of Takenouchi silt are shown in Fig. 23. It is seen that the velocity of S-wave propagation stays constant at V_s_ = 90 m/s, while the velocity of P-wave propagation becomes greater with increasing B-value. To examine the appropriate value of the skeleton Poisson’s ratio ***ν***_b_ for this silt sample, the reference curves from Eq. [22] are also drawn in Fig. 23, assuming the value of V_s_ = 90 m/s. It might be appropriate to assume that the skeleton Poisson’s ratio is approximately ***ν***_b_ = 0.4 for Takenouchi silt.

**Fig. 24:**
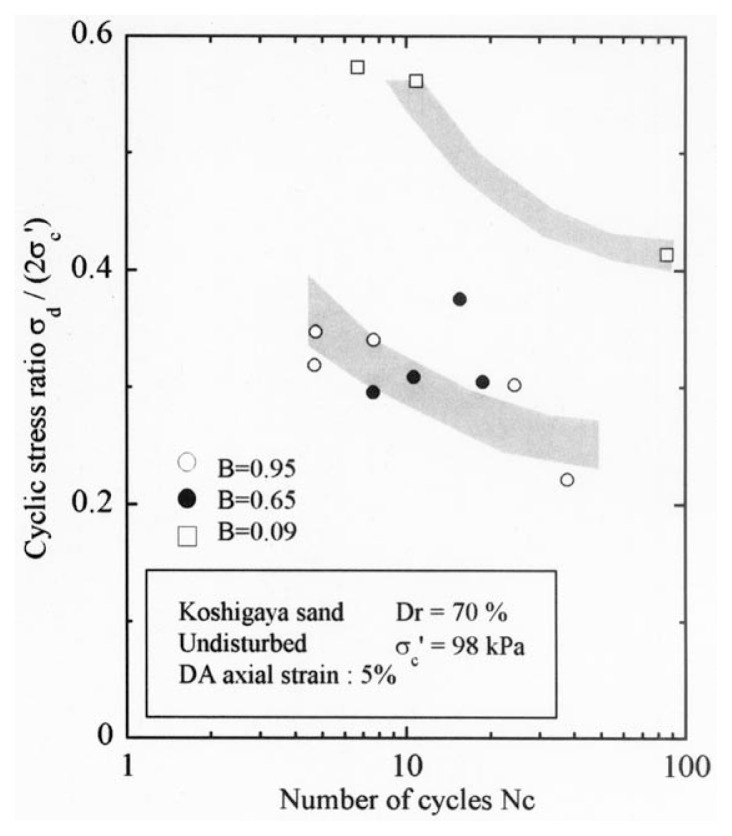
Plots of cyclic stress ratio against number of cycles (Koshigaya sand).

**Fig. 25:**
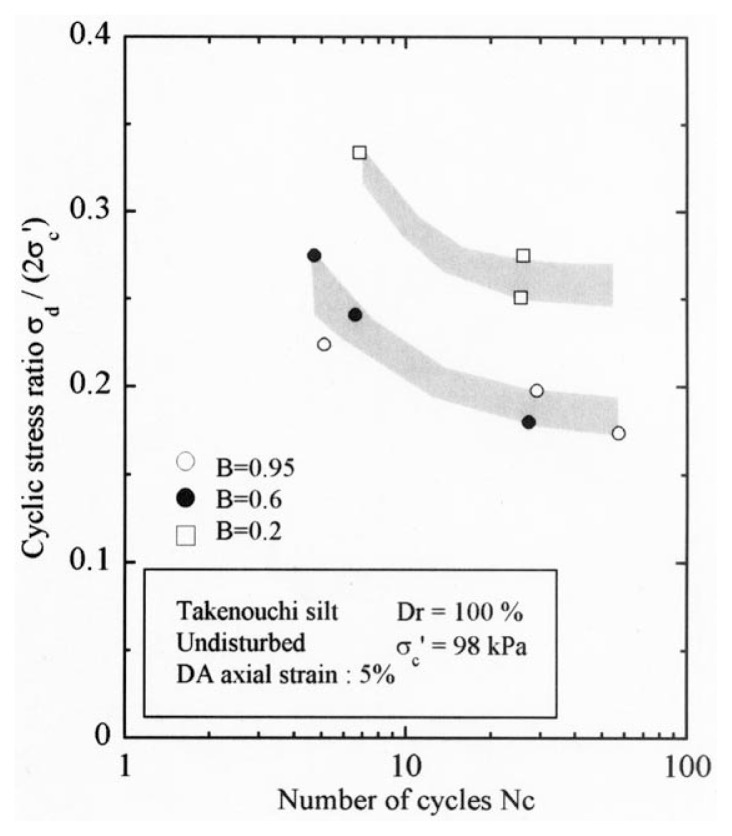
Plots of cyclic stress ratio against number of cycles (Takenouchi silt).

After the non-destructive testing of P-wave and S-wave velocity measurements, each soil specimen was subjected to undrained cyclic load with constant amplitudes of axial stress. The amplitude of the cyclic axial stress, ***σ***_d_, divided by twice the confining stress, 2***σ***_o_’, which was necessary to induce the double-amplitude (DA) axial strain of 5% is plotted against the number of cycles, N_c_, in Figs. 24 and 25.

The data for Koshigaya sand are shown in Fig. 24, and the data for Takenouchi silt are shown in Fig. 25. Noteworthy in these diagrams is the fact that the data points corresponding to the B-value range of 0.96 and 0.65 for Koshigaya sand and those corresponding to the B-value between 0.95 and 0.6 for Takenouchi silt lie around the same zone, implying that there is only a small difference in the liquefaction resistance for these soils with the B-values ranging between 0.6 and 0.95.

**Fig. 26:**
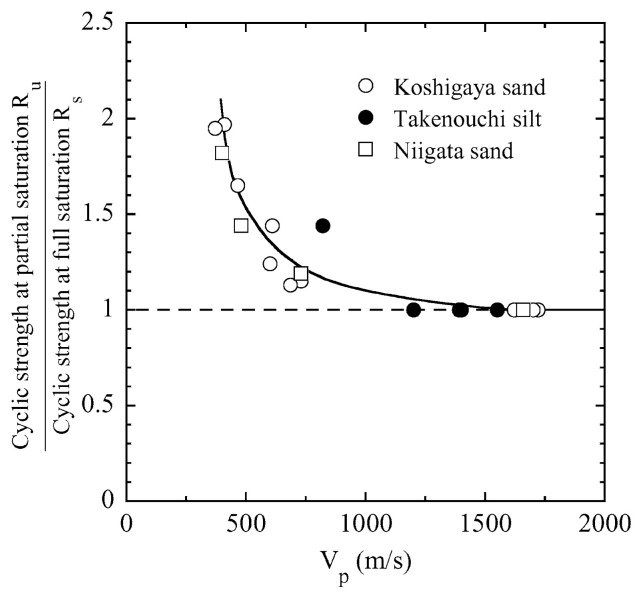
Summary plots of cyclic strength against V_p_.

On the basis of the data shown in Figs. 24 and 25, the cyclic stress ratio causing 5% DA axial strain was read off and presented in Fig. 26 in a summary form. The cyclic strength as defined above for partially saturated samples with smaller B-values was first normalized to the cyclic strength of the fully saturated samples with B ≅ 0.95, and the ratio between these two strengths is plotted in the ordinate of Fig. 26 versus the propagation velocity of the P-wave monitored in triaxial tests. For reference sake, the data in the same context obtained previously for Niigata sand are also displayed in Fig. 26. On the basis of the chart shown with a solid line in Fig. 26, the analysis of liquefaction was performed via the steps as follows:

1. First of all, the maximum horizontal acceleration was assumed. In view of the intensity of shaking on the order of V~VI in Japanese Intensity scale, the maximum acceleration was postulated to be a_max_ = 250 and 500 gals for both sites.
2. The cyclic stress ratio at 5% DA axial strain for 20 cycles was read off from Figs. 24 and 25 for saturated samples as being ***σ***_d_/(2***σ***_d_’) = 0.30 for Koshigaya site and 0.20 for Takenouchi site, respectively.
3. Entering into the solid line relation in the chart of Fig. 26 with reference to the V_p_-values measured at several depths at each site, the cyclic strength in the field deposits is determined.
4. With reference to the formula in Eq. [6], the value of ***τ***_max,_*_ℓ_*/***σ***_v_’ was evaluated with the coefficient taken jointly as C_1_·C_2_·C_5_ = 1.0.
5. The value of ***τ***_max_/***σ***_v_’ was evaluated using the relation of Eq. [4] for a_max_ = 250 and 500 gals.
6. Factor of safety was determined by the formula of Eq. [8].

**Fig. 27:**
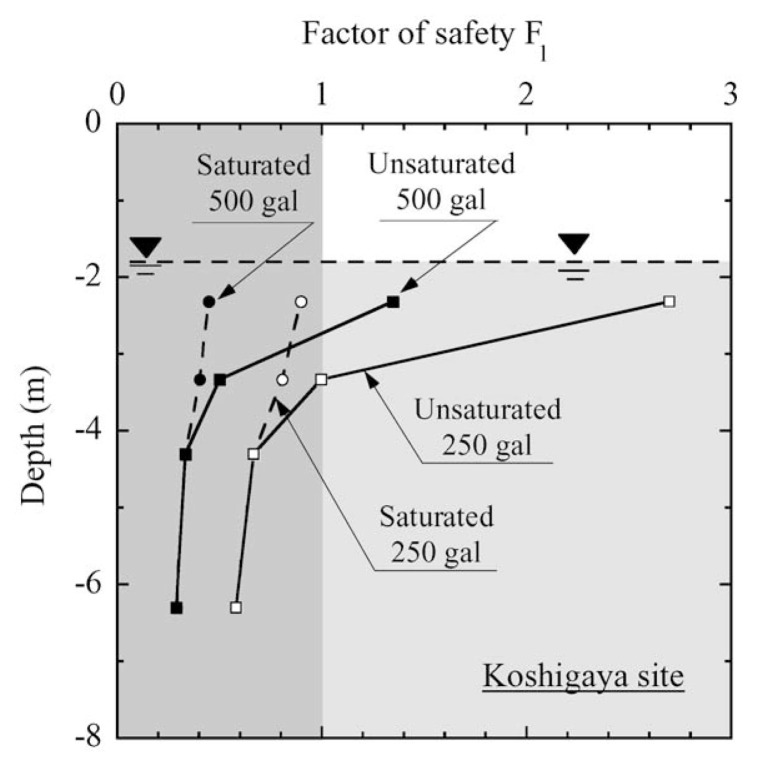
Profile of factor of safety against liquefaction with depth at Koshigaya site.

**Fig. 28:**
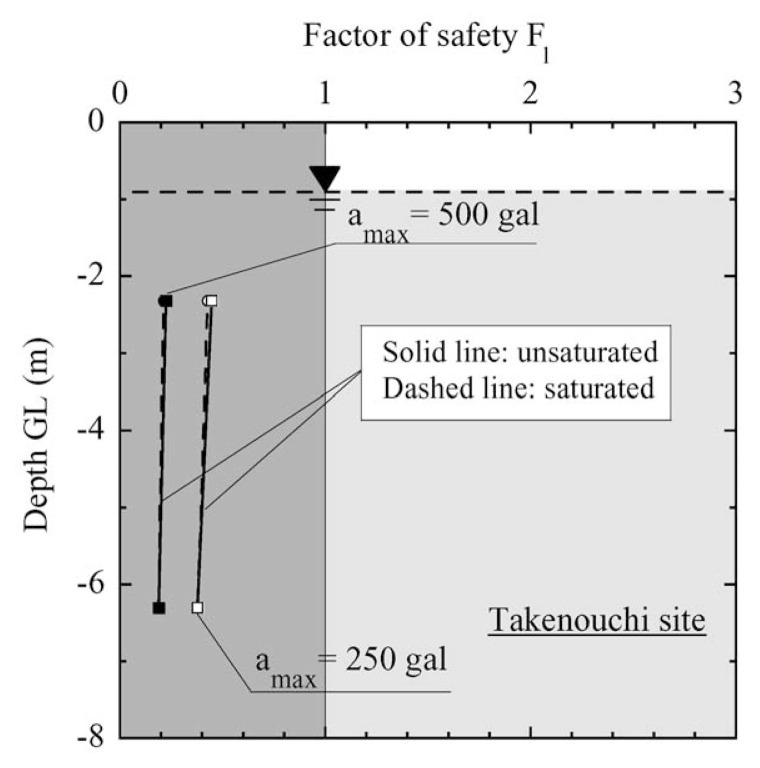
Profile of factor of safety against liquefaction with depth at Takenouchi site.

The factor of safety, F*_ℓ_*, evaluated in the above procedures is plotted in Figs. 27 and 28 versus depth of the deposit for the two sites being considered. For Koshigaya site, if the effects of partial saturation are not considered, thereby assuming the deposit to be fully saturated, the factor of safety becomes less than unity for the case of 500 gal and consequently, the liquefaction is expected to take place throughout the depth below the ground water table, bringing about considerable damage to the ground. However, if the effects of partial saturation are properly taken into account, it is anticipated that liquefaction occurs in the deposits from about 1.0 m below the ground water table for 500 gal acceleration. Thus, deleterious effects of liquefaction on structures on the ground surface are supposed to become somewhat meager and less important.

In the case of Takenouchi site in Tottori Prefecture, there is not a large difference in the liquefaction potential, no matter whether the effect of partial saturation are considered or not. In any case the factor of safety is less than unity, indicating the occurrence of liquefaction. This consequence is coincident with what happened at the time of Tottoriken-Seibu Earthquake on October 6, 2000.

## Conclusions

Results of laboratory studies have disclosed that decreasing degree of saturation tends to increase the resistance of sands to liquefaction. While the degree of saturation can be expressed in terms of the saturation ratio S_r_ or B-value in the laboratory specimens, it is practically impossible to precisely monitor these quantities in field deposits. In view of this, P-wave velocity, V_p_, was proposed as an alternative parameter which could be used for quantifying the degree of saturation. The usage of P-wave velocity has several advantages; namely, it can be monitored both in-situ and in the laboratory, and it is precise enough to correlate a meaningful change in the saturation ratio S_r_ or B-value with the cyclic resistance of partly saturated sand. Because of its non-destructive nature of the tests, undisturbed state of a sample from in-situ deposit can be preserved when testing it in the laboratory to determine the cyclic strength producing large strains at failure.

With these views in mind, an additional setup was attached to the conventional type of cyclic triaxial test apparatus so that the velocities of both P- and S-waves can be monitored in the laboratory without disturbing the samples prior to the cyclic loading tests. Since the B-value was measured in the laboratory tests together with V_p_ and V_s_, it was possible to obtain a set of test data for plotting the ratio V_p_/V_s_ versus the B-value and to determine the skeleton Poisson’s ratio ***ν***_b_. The results of the tests showed that the skeleton Poisson’s ratio was ***ν***_b_ ≅ 0.35.

After measurements of P- and S-wave velocities, cyclic loading tests were performed on the same sand sample and the cyclic stress ratio causing liquefaction was determined. As a result of the tests, the resistance to liquefaction was found to increase with decreasing B-value. When the B-value drops to zero with the saturation ratio of S_r_ = 90%, the cyclic strength becomes twice as much as that at the full saturation with B = 0.95.

To examine applicability of the test procedures proposed, two sites were chosen and a set of in-situ tests was conducted including the SPT-sounding, undisturbed sampling and velocity loggings. From the results of the velocity logging tests, it was found that the imperfectly saturated soil layers exist immediately below the ground water table where the velocity of P-wave propagation was of the order of 700~1300 m/s. Then, laboratory tests were conducted on undisturbed soil specimens recovered from the field deposits. By using the relation between V_p_ and the cyclic strength thus obtained, the influence of partial saturation on the liquefaction resistance of soils was evaluated and incorporated into the analysis of liquefaction for these two sites. The results of the analysis revealed that, at one site being considered, the P-wave velocity was on the order of 700 m/sec through the depth of 2 m below the ground water table and therefore the liquefaction resistance was found to be stronger in this depth range of the deposits. At another site, the liquefaction was found to occur anyway, no matter whether the soil is partly or fully saturated.
