# Errata to “Cyclic strength of imperfectly saturated sands and analysis of liquefaction” [Proc. Jpn. Acad., Ser. B 80, No. 8, 372–391 (2004)]

**Published:** 2004-09-01

**Authors:** Kenji Ishihara, Yoshimichi Tsukamoto

In this paper, the phrases should be corrected as follows:

(page 377, left column, line 3)

For “coefficients jointly takes a value of C_1_C_2_C_5_ ≅ 0.65.”

Read “coefficients jointly takes a value of C_1_C_2_C_5_ ≅ 1.0.”

(page 389, left column, line 3 from bottom)

For “jointly as C_1_·C_2_·C_5_ = 0.65.”

Read “jointly as C_1_·C_2_·C_5_ = 1.0.”

